# JSCSNCP-LMA: a method for predicting the association of lncRNA–miRNA

**DOI:** 10.1038/s41598-022-21243-y

**Published:** 2022-10-11

**Authors:** Bo Wang, Xinwei Wang, Xiaodong Zheng, Yu Han, Xiaoxin Du

**Affiliations:** College of Computer and Control Engineering, Qiqihar University, Qiqihar, 161006 People’s Republic of China

**Keywords:** Computational biology and bioinformatics, Computational models

## Abstract

Non-coding RNAs (ncRNAs) have long been considered the "white elephant" on the genome because they lack the ability to encode proteins. However, in recent years, more and more biological experiments and clinical reports have proved that ncRNAs account for a large proportion in organisms. At the same time, they play a decisive role in the biological processes such as gene expression and cell growth and development. Recently, it has been found that short sequence non-coding RNA(miRNA) and long sequence non-coding RNA(lncRNA) can regulate each other, which plays an important role in various complex human diseases. In this paper, we used a new method (JSCSNCP-LMA) to predict lncRNA–miRNA with unknown associations. This method combined Jaccard similarity algorithm, self-tuning spectral clustering similarity algorithm, cosine similarity algorithm and known lncRNA–miRNA association networks, and used the consistency projection to complete the final prediction. The results showed that the AUC values of JSCSNCP-LMA in fivefold cross validation (fivefold CV) and leave-one-out cross validation (LOOCV) were 0.9145 and 0.9268, respectively. Compared with other models, we have successfully proved its superiority and good extensibility. Meanwhile, the model also used three different lncRNA–miRNA datasets in the fivefold CV experiment and obtained good results with AUC values of 0.9145, 0.9662 and 0.9505, respectively. Therefore, JSCSNCP-LMA will help to predict the associations between lncRNA and miRNA.

## Introduction

NcRNAs are a class of RNAs that don’t have the function of translating proteins in organisms^1–3^. Therefore, ncRNAs have always been neglected by biological researchers. With the progress of science and technology, gene detection technology is also developing. Researchers have found that RNAs don’t participate in protein coding account for about 98% of RNA in organisms^4^. As a result, researchers are increasingly interested in ncRNAs. Studies have found that ncRNAs can be divided into many types, including lncRNA, miRNA, circRNA, and snRNA^5,6^. MiRNAs are a class of ncRNAs with a short sequence of 18–25 nucleotides in length, while lncRNAs are a class of ncRNAs with a length of more than 200 nucleotides^7–11^. Experiments have found that lncRNAs play an important role in various biological processes such as transcription, translation and differentiation^12–16^. Meanwhile, mutations and dysregulations of these lncRNAs have also been shown to have complex relationships with many human complex diseases^17^, such as lung cancer^18^, AIDS^19^, cardiovascular disease^20^, Alzheimer's disease (AD)^21^, and diabetes^22^. For another example, lncRNA HOTAIR, PCA3 and H19 have been treated as potential biomarkers of hepatocellular carcinoma recurrence^23^, prostate cancer aggressiveness^24^ and breast cancer detection, respectively^25^. Similarly, miRNAs also play a key role in the differentiation, proliferation and apoptosis of biological cells^26–30^. Correspondingly, more and more miRNAs have been proved to have an impact on the occurrence of certain diseases. For example, in the midbrain of patients with Parkinson’s disease, miRNAs were regarded as a regulator to the maturation and function of midbrain dopaminergic neurons^31^. Overexpression of mir-128 in glioma cells was proved to inhibit cell proliferation^31,32^. Furthermore, mir-375 could regulate insulin secretion^33^; the miR-1 was involved in heart development; deletion of miRNA-1–2 interrupted the regulation of carcinogenesis^34,35^.

Recently, studies have shown that some specific lncRNAs and miRNAs can be detected in serum and blood of some cancer patients^36,37^. At the same time, lncRNAs and miRNAs can interact with each other in some diseases, which jointly affects the occurrence of human diseases^38,39^. For example, in the COVID-19 study, after comparing the transcriptomic data from different patient groups, researchers observed that COVID-19 patients had abnormally expressed mRNA and lncRNA when they were admitted to the ICU. Shaath and Alajez suggested that further studies on the identification and role of these mRNA and lncRNA-based biomarkers, as well as their impact on the onset and severity of COVID-19, which could play a crucial role in patient stratification and help select appropriate treatment options^40^. Therefore, the research on the associations between lncRNAs and miRNAs has become a research boom. However, it is very complicated to verify the associations between lncRNAs and miRNAs only through biological experiments. In order to improve the research efficiency of biological researchers, researchers in the computer field use existing biological data to analyze and predict the unknown associations between lncRNAs and miRNAs.

Before that, some prediction models of ncRNA-disease were well-established and had good results. For example, Chen et al. developed a reliable computational tool of LRLSLDA to predict novel human lncRNA-disease associations based on the assumption that similar diseases could tend to be related with functionally similar lncRNAs. This model was mainly based on a semi-supervised learning framework of Laplacian Regularized Least Squares^41^, which integrated known disease-lncRNA associations and lncRNA expression profile. In 2015, based on the assumption that similar diseases could tend to be associated with lncRNAs with similar functions, Chen et al. further developed two novel lncRNA functional similarity calculation models (LNCSIM)^42^. In the model of LNCSIM, disease semantic similarity was first calculated based on the directed acyclic graph (DAG) which represented the relationships among different diseases. Then, lncRNA functional similarity was further obtained by calculating the semantic similarity between their associated disease groups. Yang et al. implemented a propagation algorithm on the coding-non-coding gene-disease bipartite network to infer potential lncRNA-disease associations^43^. The coding-non-coding gene-disease bipartite network was constructed by integrating known lncRNA-disease associations and gene-disease associations. Chen developed a computational model of KATZLDA to identify potential lncRNA-disease associations by known lncRNA-disease associations and various similarity measures of diseases and lncRNAs^44^. Considering the limitations of traditional Random Walk with Restart (RWR), the model of Improved Random Walk with Restart for LncRNA-Disease Association prediction (IRWRLDA^45^) was developed by Chen et al. to predict novel lncRNA-disease associations by integrating known lncRNA-disease associations, disease semantic similarity, and various lncRNA similarity measures.

Meanwhile, Chen et al.^46^ proposed a novel computational model of Within and Between Score for MiRNA-Disease Association prediction (WBSMDA) by incorporating miRNA functional similarity, disease semantic similarity, miRNA-disease associations and Gaussian interaction profile kernel similarity for diseases and miRNAs. Li et al.^47^ developed a matrix completion for MiRNA-disease association prediction model (MCMDA). This model used the Lagrange multiplier method to update the adjacency matrix of known miRNA-disease associations and further predict potential associations. Chen et al.^48^ further proposed a model called graph regression for MiRNA-disease association prediction. The model carried out graph regression in three spaces, including association space, miRNA similarity space and disease similarity space. Then, Chen et al.^49^ put forward Inductive Matrix Completion for MiRNA-Disease Association prediction (IMCMDA), which could apply to new diseases without known miRNAs. Furthermore, Chen et al.^50^ developed another prediction miRNA-disease association prediction model of Bipartite Network Projection for MiRNA-Disease Association prediction (BNPMDA). This model first constructed the bias ratings for miRNAs and diseases based on three networks, including the known miRNA-disease association network, the disease similarity network and the miRNA similarity network. Then bipartite network recommendation algorithm was implemented to reveal potential miRNA-disease associations. Chen et al.^51^, proposed a novel computational method named Ensemble of Decision Tree based MiRNA-Disease Association prediction (EDTMDA), which innovatively built a computational framework integrating ensemble learning and dimensionality reduction. The model adopted ensemble learning strategy that integrated multiple classifiers (base learners) to get final prediction results. Then, Chen et al.^52^, developed the model of deep-belief network for miRNA-disease association prediction (DBNMDA). DBNMDA innovatively utilized the information of all miRNA-disease pairs during the pre-training process. This step could reduce the impact of too few known associations on prediction accuracy to some extent. In addition, Chen et al.^53^, proposed a new computational model named Neighborhood Constraint Matrix Completion for MiRNA-Disease Association prediction (NCMCMDA) to predict potential miRNA-disease associations. The model innovatively integrated neighborhood constraint with matrix completion, which provided a novel idea of utilizing similarity information to assist the prediction. After the recovery task was transformed into an optimization problem, this model solved it with a fast iterative shrinkage-thresholding algorithm.

However, the current lncRNA–miRNA association prediction models mainly use machine learning algorithms. Huang et al.^54^ proposed a method named EPLMI, which relied on the assumption that lncRNAs having similar expression profiles were prone to associate with a cluster of miRNAs that had similar expression profiles. However, a new question had arisen as to how to use the expression profile of ncRNAs to define the similarity between them. The EPLMI model calculated the similarity using the Person correlation coefficient, which was basically consistent with the hypothetical ncRNAs feature similarity score of each element pair. Nevertheless, the method still had some problems due to the nature of its mechanism. Liu et al.^55^ proposed the LMFNRLMI model, which utilized the strongest neighborhood relationship and established a neighborhood matrix to predict the lncRNA–miRNA association by using the K nearest neighbor method. However, there was still a lack of high-performance and high-precision models to predict potential lncRNA–miRNA associations. At the same time, Huang et al.^56^ developed a novel group preference Bayesian collaborative filtering model (GBCF), which picked up a top-k probability ranking list for an individual miRNA or lncRNA based on known lncRNA–miRNA interaction network. However, the Bayesian classifier needed to have negative samples to improve its performance. There were no negative samples in the lncRNA–miRNA association studies, and a random selection of positional association as negative samples would affect the prediction performance. A sequence-derived linear neighborhood propagation method (SLNPM) to predict lncRNA–miRNA associations was proposed by Zhang et al.^57^. Firstly, miRNA–miRNA similarity and lncRNA–lncRNA similarity were calculated by using miRNA sequence and lncRNA sequence and the known lncRNA–miRNA associations. Secondly, the integrated lncRNA similarity-based graph and the integrated miRNA similarity-based graph were respectively constructed, and the label propagation processes were respectively implemented on two graphs to score lncRNA–miRNA pairs. Finally, the averages of their outputs were adopted as final predictions. However, these methods still have some limitations, which will inspire us to develop better models. In the association prediction of between lncRNA and miRNA, the focus and difficulty of the next step is to further reduce the dependence of the model on the quality of the lncRNA and miRNA similarity matrix, pay more attention to the difference of correlation strength, reduce the complexity of model calculation, and avoid the prediction model to bias towards some well-studied lncRNAs or miRNAs. In the future development of lncRNA–miRNA association prediction, cloud computing can further make it possible to mine complex large-scale information. It can further explore the deep correlation between lncRNAs and miRNAs related indicators, so as to find out that the joint action of lncRNAs and miRNAs leads to the occurrence of diseases. Therefore, the diseases can be accurately predicted before they are formed, so as to carry out manual intervention as early as possible, make a more accurate description of the degree of disease, and find a series of changes in the body to form the root (lncRNAs and miRNAs) of the disease, so as to achieve accurate and efficient treatment.

In this paper, in order to more effectively predict potential associations between lncRNA and miRNA, we proposed a new computational method called Network Consistency projection for the Human LncRNA–miRNA Association (JSCSNCP-LMA). JSCSNCP-LMA achieved excellent prediction performance by using Jaccard similarity algorithm, self-correcting spectral clustering similarity algorithm, cosine similarity algorithm and known lncRNA–miRNA association network to predict lncRNA–miRNA of unknown associations. There are three advantages to this method. First of all, the algorithm in our prediction model is relatively simple and has no complex parameters. And the algorithm can also get good prediction results. Furthermore, our method could be also used for other association prediction, which has good expansibility. Last but not least, we can use lncRNA–miRNA association prediction to further study lncRNA-disease association prediction or miRNA-disease association prediction, so as to improve the accuracy of lncRNA-disease association prediction or miRNA-disease association prediction. To demonstrate the prediction performance of the JSCSNCP-LMA, LOOCV and fivefold CV were used to test the model. The results showed that the AUC values of the proposed JSCSNCP-LMA were 0.9268 and 0.9145, respectively.

## Datasets and methods

### Datasets

For lncRNA, miRNA, and lncRNA–miRNA interactions data, there are many open-source datasets available for online download. For example, miRBase^58^, miRmine^59^, NONCODE^60^, and lncRNASNP^61^. We obtained three different datasets from different databases in order to verify the accuracy of our experiment. The specific operations are as follows. Firstly, we downloaded data from lncRNASNP, and obtained 8091 experimentally verified lncRNA–miRNA interactions. After removing duplicated associations, we obtained 275 miRNAs and 780 lncRNAs. Then, we collected lncRNAs’ sequences from NONCODE and miRNAs’ sequences from miRbase. We finally obtained 417 lncRNAs and 265 miRNAs, which could be used as our Data 1. Secondly, we downloaded and cleaned the starBasev2.0^62^ database on the ENCORI (open source platform). After processing, we obtained 1089 lncRNAs and 246 miRNAs, which could be used as our Data 2. Thirdly, we obtained the lncRNA–miRNA interactions from the known lncRNASNP2 database. After processing, we finally obtained 8634 lncRNA–miRNA interactions, including 468 lncRNAs and 262 miRNAs, which could be used as our Data 3. Three datasets were finally obtained, as shown in Table 1 below.

**Table 1:** Data sheet.

Name	LncRNAs	MiRNAS	Interactions
DATA 1	417	265	2272
DATA 2	1089	246	9086
DATA 3	468	262	8634

In this paper, we let $L=\left\{{l}_{1},{l}_{2},{l}_{3},\dots ,{l}_{r}\right\}$ and $M=\left\{{m}_{1},{m}_{2},{m}_{3},\dots ,{m}_{n}\right\}$, which represented the set of *r* lncRNAs and *n* miRNAs. We defined adjacency matrix ${\varvec{Y}}$ to represent the relationship between lncRNA and miRNA interactions. If $lncRNA {l}_{i}$ was verified to interact with $miRNA {m}_{j}$, then $Y (i, j)$ was assigned 1, otherwise 0. We let $Y\left({l}_{i}\right)=\left\{{l}_{1},{l}_{2},{l}_{3},\dots ,{l}_{r}\right\}$ and $Y\left({m}_{j}\right)=\left\{{m}_{1},{m}_{2},{m}_{3},\dots ,{m}_{n}\right\}$, which represented the row *i*-vector of matrix ${\varvec{Y}}$ and the column *j*-vector of matrix ${\varvec{Y}}$. $Y({l}_{i})$ and $Y({m}_{j})$ represented the interactions of $lncRNA {l}_{i}$ and $miRNA {m}_{j}$, respectively. Matrix ${\varvec{Y}}$ is defined as follows:

1 $$Y(i, j) = \left\{\begin{array}{l} 0 \; miRNA \; m(j) \; has \; no \; association \; with \; lncRNA \; l(i)\\ 1 \; miRNA \; m(j) \; has \; association \; with \; lncRNA \; l(i)\end{array}\right.$$

## Methods

### Cosine similarity for lncRNA and miRNA

Previously, cosine similarity algorithm has been widely used by researchers in the collaborative filtering recommendation algorithm^63,64^. Recently, Gaussian distribution kernel similarity algorithm has been widely used to calculate the similarity of individual biomolecules in human body. However, its performance is lower than the cosine similarity algorithm. Therefore, in this paper we decided to use cosine similarity as a complementary dimension of lncRNA and miRNA similarity. The principle of lncRNA cosine similarity was based on the assumption that if $lncRNA {l}_{i}$ and $lncRNA {l}_{j}$ were similar to each other, then in the lncRNA–miRNA association matrix, binary vector $Y({l}_{i})$ and binary vector $Y({l}_{j})$ should be similar to each other. The same assumption should also be true for miRNA. Based on known lncRNA–miRNA associations data, the cosine similarity matrix ***CL*** of lncRNA is calculated as follows:

2 $$CL\left({l}_{i},{l}_{j}\right)= \frac{Y\left({l}_{i}\right)\cdot Y\left({l}_{j}\right)}{\Vert Y\left({l}_{i}\right)\Vert \Vert Y\left({l}_{j}\right)\Vert },$$

3 $$CL ={((CL({l}_{i},{l}_{j}))}_{r*r}.$$

The binary vector $Y({l}_{i})$ indicates whether there is an association between $lncRNA {l}_{i}$ and each miRNA (the row *i* of the adjacency matrix ${\varvec{Y}}$, 1 if ${l}_{i}$ is related to miRNA, otherwise 0). Meanwhile, $CL({l}_{i},{l}_{j})$ is the cosine similarity of between $lncRNA {l}_{i}$ and $lncRNA {l}_{j}$. The ${\varvec{C}}{\varvec{L}}$ is the lncRNA cosine similarity matrix.

Similarly, the cosine similarity of $miRNA {m}_{i}$ and $miRNA {m}_{j}$ is calculated as follows:

4 $$CM\left({m}_{i},{m}_{j}\right)= \frac{Y\left({m}_{i}\right)\cdot Y\left({m}_{j}\right)}{\Vert Y\left({m}_{i}\right)\Vert \Vert Y\left({m}_{j}\right)\Vert },$$

5 $$CM ={((CM({m}_{i},{m}_{j}))}_{n*n}.$$

The binary vector $Y({m}_{i})$ indicates whether there is an association between $miRNA {m}_{i}$ and each lncRNA (the column *j* of adjacency matrix ${\varvec{Y}}$, 1 if ${m}_{j}$ is related to lncRNA, otherwise 0). Meanwhile, $CM({m}_{i},{m}_{j})$ is the cosine similarity of between $miRNA {m}_{i}$ and $miRNA {m}_{j}$. The ${\varvec{C}}{\varvec{M}}$ is the miRNA cosine similarity matrix.

### Jaccard similarity for lncRNA and miRNA

Recently, Jaccard similarity coefficient has been widely used in recommendation algorithms^65^. Meanwhile, it has been used by researchers to predict the associations of biological factors, because it is mainly used to compare the similarity between limited sample sets, and it does not consider the potential value size in the vector. In this paper, we used Jaccard similarity to calculate the similarity of lncRNA and miRNA respectively. The Jaccard similarity matrix ${\varvec{J}}{\varvec{L}}$ of lncRNA is calculated as follows:

6 $$JL\left({l}_{i},{l}_{j}\right)= \frac{\left|Y\left({l}_{i}\right)\cap \left.Y\left({l}_{j}\right)\right|\right.}{\left|Y\left({l}_{i}\right)\cup \left.Y\left({l}_{j}\right)\right|\right.},$$

7 $$JL ={((JL({l}_{i},{l}_{j}))}_{r*r},$$

where $Y({l}_{i})$ and $Y({l}_{j})$ represent the number of miRNAs sets associated with $lncRNA {l}_{i}$ and $lncRNA {l}_{j}$, respectively. The ${\varvec{J}}{\varvec{L}}$ is the lncRNA Jaccard similarity matrix. Similar to lncRNA, the Jaccard similarity between $miRNA {m}_{i}$ and $miRNA {m}_{j}$ can be calculated as follows:

8 $$JM\left({m}_{i},{m}_{j}\right)= \frac{\left|Y\left({m}_{i}\right)\cap \left.Y\left({m}_{j}\right)\right|\right.}{\left|Y\left({m}_{i}\right)\cup \left.Y\left({m}_{j}\right)\right|\right.},$$

9 $$JM ={((JM({m}_{i},{m}_{j}))}_{n*n},$$

where $Y({m}_{i})$ and $Y({m}_{j})$ represent the number of miRNAs sets associated with $miRNA {m}_{i}$ and $miRNA {m}_{j}$, respectively. The ${\varvec{J}}{\varvec{M}}$ is the miRNA Jaccard similarity matrix.

### Self-tuning spectral clustering similarity for lncRNA and miRNA

After Jaccard similarity calculation, the similarity matrix we obtained was still relatively sparse. To improve the accuracy of the experiment, we used self-tuning spectral clustering similarity algorithm to fill the sparse matrix^66^. Spectral clustering is one of the methods of clustering, which can deal with complex and diverse structured data. It does not require an explicit model for estimating the data distribution, but rather performs a spectral analysis of the point-to-point similarity matrix. In this paper, we concluded that similar lncRNAs were related to similar function miRNAs, so we used self-tuning spectral clustering similarity algorithm to calculate the similarity between $lncRNA {l}_{i}$ and $lncRNA {l}_{j}$. We represented the rows *i* and *j* vectors of the matrix ${\varvec{Y}}$ as $VL({l}_{i})$ and $VL({l}_{j})$, respectively. Then, the self-tuning spectral clustering similarity of lncRNAs can be calculated as follows:

10 $$SL({l}_{i},{l}_{j}) = \left\{\begin{array}{ll}exp\left(\frac{{-\Vert VL({l}_{i})-VL({l}_{j})\Vert }^{2}}{{\delta }_{i}{\cdot \delta }_{j}}\right) , & i\ne j \\ 0 , & i=j,\end{array}\right.$$

11 $${\delta }_{i} =\Vert VL({l}_{i})-VL({l}_{iK})\Vert ,$$

where, $VL({l}_{iK})$ is the *K* th adjacent point of the $VL({l}_{i})$ sample point, and here we default ***K*** value is 5.

12 $$SL ={((SL({l}_{i},{l}_{j}))}_{r*r},$$

where, ${\varvec{S}}{\varvec{L}}$ is the self-tuning spectral cluster similarity matrix of lncRNAs.

Similarly, we represented the column *i* and *j* vectors of the matrix ${\varvec{Y}}$ as $VM({m}_{i})$ and $VM({m}_{j})$, respectively. The self-tuning spectral clustering similarity of miRNAs can be calculated as follows:

13 $$SM({m}_{i},{m}_{j}) = \left\{\begin{array}{ll}exp\left(\frac{{-\Vert VM({m}_{i})-VM({m}_{j})\Vert }^{2}}{{\delta }_{i}{\cdot \delta }_{j}}\right) , & i\ne j \\ 0 , & i=j,\end{array}\right.$$

14 $${\delta }_{i} =\Vert VM({m}_{i})-VM({m}_{iK})\Vert ,$$

where, $VM({m}_{iK})$ is the ***K*** th adjacent point of the $VM({m}_{i})$ sample point, and here we default ***K*** value is 5.

15 $$SM ={((SM({m}_{i},{m}_{j}))}_{n*n},$$

where, ${\varvec{S}}{\varvec{M}}$ is the self-tuning spectral cluster similarity matrix of miRNAs.

### Integrated lncRNA similarity and miRNA similarity

After the above steps, we obtained the cosine similarity matrix, Jaccard similarity matrix, and self-tuning spectral cluster similarity matrix of lncRNAs and miRNAs. Then, we integrated various similarity matrices to obtain a more complete similarity matrix. First, we combined the Jaccard similarity matrix of lncRNAs with the self-tuning spectral cluster similarity matrix of lncRNAs. The integrated similarity matrix ***JSL*** of the lncRNAs can be calculated, as follows:

16 $$JSL({l}_{i},{l}_{j}) =\left\{\begin{array}{ll}\frac{JL({l}_{i},\,{l}_{j})+SL({l}_{i},{l}_{j})}{2} , & \,JL({l}_{i},{l}_{j}) \ne 0\\ SL({l}_{i},{l}_{j}) , & \,JL({l}_{i},{l}_{j}) = 0,\end{array}\right.$$

17 $$JSL ={((JSL({l}_{i},{l}_{j}))}_{r*r},$$

where, we used the self-tuning spectral cluster similarity to supplement the Jaccard similarity. If $JL({l}_{i},\,{l}_{j})$ was 0, it was filled directly by $SL({l}_{i},{l}_{j})$, otherwise the mean was taken, so that the matrix was more complete to make the sparse matrix dense and improve the accuracy of the experimental results. Second, we combined the Jaccard similarity matrix of miRNAs with the self-tuning spectral cluster similarity matrix of miRNAs. The integrated similarity matrix ***JSM*** of the miRNAs can be calculated, as follows:

18 $$JSM({m}_{i},{m}_{j}) =\left\{\begin{array}{ll}\frac{JM({m}_{i},{m}_{j})+SM({m}_{i},{m}_{j})}{2} , & \, JM({m}_{i},{m}_{j}) \ne 0\\ SM({m}_{i},{m}_{j}) , & \,JM({m}_{i},{m}_{j}) = 0,\end{array}\right.$$

19 $$JSM ={((JSM({m}_{i},\,{m}_{j}))}_{n*n}.$$

To better supplement the dimension of the similarity matrix, we would re-integrate the cosine similarity with the new integrated similarity matrix. Specifically, if $lncRNA {l}_{i}$ and $lncRNA {l}_{j}$ had no common associated miRNA in the adjacency matrix ${\varvec{Y}}$, then the value between them was 0 in the cosine similarity matrix ${\varvec{C}}{\varvec{L}}$. Therefore, when the $lncRNA {l}_{i}$ and $lncRNA {l}_{j}$ had no similarity scores in the ${\varvec{C}}{\varvec{L}}$, we directly used the ${\varvec{J}}{\varvec{S}}{\varvec{L}}$ similarity score between them as the integrated similarity score. If $lncRNA {l}_{i}$ and $lncRNA {l}_{j}$ had similar scores in ${\varvec{C}}{\varvec{L}}$, then we integrated the similarity scores in ${\varvec{C}}{\varvec{L}}$ and ${\varvec{J}}{\varvec{S}}{\varvec{L}}$ as the final similarity scores. We conducted experiments on the weight parameters of the integration process and found that 0.5 was the best integration. The integrated similarity matrix ***JSCL*** of the lncRNAs can be calculated, as follows:

20 $$JSCL({l}_{i},{l}_{j}) =\left\{\begin{array}{ll}\frac{JSL({l}_{i},{l}_{j})+CL({l}_{i},{l}_{j})}{2} , & \,CL({l}_{i},{l}_{j}) \ne 0\\ JSL({l}_{i},{l}_{j}) , & \,CL({l}_{i},{l}_{j}) = 0,\end{array}\right.$$

21 $$JSCL ={((JSCL({l}_{i},{l}_{j}))}_{r*r}.$$

Similarly, if the $miRNA {m}_{i}$ and $miRNA {m}_{j}$ had no common associated lncRNA in the adjacency matrix ${\varvec{Y}}$, then the value between them was 0 in the cosine similarity matrix ${\varvec{C}}{\varvec{M}}$. Therefore, when the $miRNA {m}_{i}$ and $miRNA {m}_{j}$ had no similarity scores in the ${\varvec{C}}{\varvec{M}}$, we directly used the ${\varvec{J}}{\varvec{S}}{\varvec{M}}$ similarity score between them as the integrated similarity score. If $miRNA {m}_{i}$ and $miRNA {m}_{j}$ had similar scores in ${\varvec{C}}{\varvec{M}}$, then we integrated the similarity scores in ${\varvec{C}}{\varvec{M}}$ and ${\varvec{J}}{\varvec{S}}{\varvec{M}}$ as the final similarity scores. The integrated similarity matrix ***JSCM*** of the miRNAs can be calculated, as follows:

22 $$JSCM({m}_{i},{m}_{j}) =\left\{\begin{array}{ll}\frac{JSM({m}_{i},{m}_{j})+CM({m}_{i},{m}_{j})}{2} , & CM({m}_{i},{m}_{j}) \ne 0\\ JSM({m}_{i},{m}_{j}) , & CM({m}_{i},{m}_{j}) = 0,\end{array}\right.$$

23 $$JSCM ={((JSCM({m}_{i},{m}_{j}))}_{n*n}.$$

## Network consistent projection of the human

### LncRNA–miRNA association

**Figure 1:**
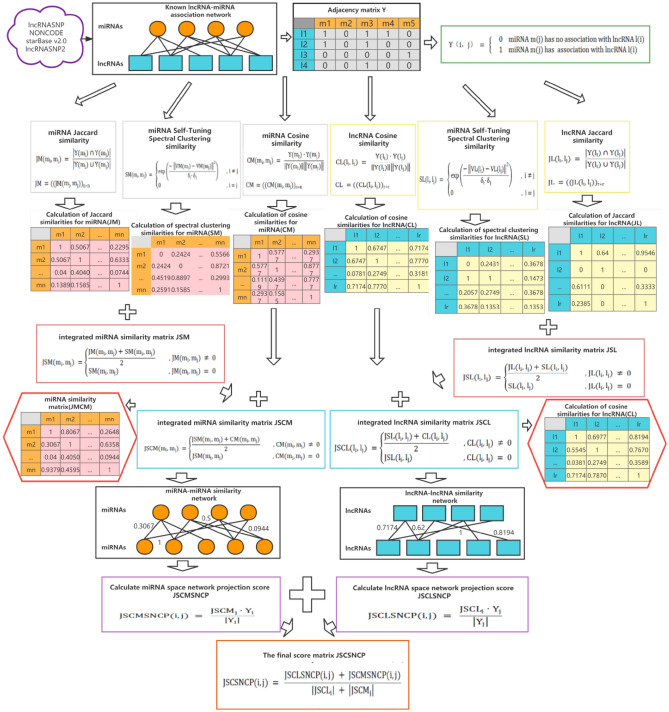
Flow chart of JSCSNCP-LMA applied to lncRNAs–miRNAs association prediction. The JSCSNCP-LMA including four steps: First, construct the lncRNA–miRNA association matrix. Second, calculate the cosine similarity matrix of lncRNA and miRNA, Jaccard similarity matrix of lncRNA and miRNA and self-tuning spectral cluster similarity matrix of lncRNA and miRNA. Third, integrate the above matrix to obtain the comprehensive similarity matrix of lncRNA and miRNA. Finally, the final prediction score matrix is calculated by the network consistency projection.

In our study, network consistency projection predicted potential lncRNA–miRNA associations through heterogeneous networks. The heterogeneous networks included the known lncRNA–miRNA association networks, the integrated lncRNAs similarity network (${\varvec{J}}{\varvec{S}}{\varvec{C}}{\varvec{L}}$), and the integrated miRNAs similarity network (${\varvec{J}}{\varvec{S}}{\varvec{C}}{\varvec{M}}$). JSCSNCP-LMA was divided into two parts: lncRNAs spatial consistency projection score and miRNAs spatial consistency projection score. The flow chart of the JSCSNCP-LMA method is shown in Fig. 1.

### LncRNA space consistency projection score

Network consistency projection refers to the higher similarity score between $lncRNA {l}_{i}$ and other lncRNA (including $lncRNA {l}_{i}$ itself) in the integrated lncRNA similarity matrix (${\varvec{J}}{\varvec{S}}{\varvec{C}}{\varvec{L}}$), more lncRNAs related to $miRNA {m}_{j}$, and the high spatial similarity of $lncRNA {l}_{i}$ with $miRNA {m}_{j}$. In the adjacency matrix ${\varvec{Y}}$***,*** the values of the unknown associations are all 0; but in fact, these unknown associations are uncertain. Therefore, we replaced each of them with one small positive integer $\delta$, and the value of $\delta$ was set as 10–30. This approach prevents the denominator from being 0 and will not affect the result of the calculation. The lncRNA space consistency projection scores are calculated as follows:

24 $$JSCLSNCP\left(i,j\right)= \frac{{JSCL}_{i}\cdot {Y}_{j}}{\left|{Y}_{j}\right|},$$

where, ${JSCL}_{i}$ is the *i* th row of the integrated lncRNAs similarity matrix ${\varvec{J}}{\varvec{S}}{\varvec{C}}{\varvec{L}}$, which represents the similarity of $lncRNA {l}_{i}$ with all lncRNAs. ${Y}_{j}$ is the *j*th column of the adjacency matrix ${\varvec{Y}}$, which represents the association of $miRNA {m}_{j}$ with all lncRNAs. $\left|{Y}_{j}\right|$ is the length of the vector ${Y}_{j}$, which is also the modules of the vector. $JSCLSNCP(i,j)$ represents the network consistency projection score of ${JSCL}_{i}$ on ${Y}_{j}$. Specifically, if the angle of the network projection of ${JSCL}_{i}$ on ${Y}_{j}$ is smaller, then the value of $JSCLSNCP(i,j)$ is larger.

### MiRNA space consistency projection score

Similarly, the miRNA space consistency projection scores are calculated as follows:

25 $$JSCMSNCP\left(i,j\right)= \frac{{JSCM}_{j}\cdot {Y}_{i}}{\left|{Y}_{i}\right|},$$

where, ${JSCM}_{j}$ is the *j* th column of the integrated miRNAs similarity matrix ***JSCM***, which represents the similarity of $miRNA {m}_{j}$ with all miRNAs. ${Y}_{i}$ is the *i*th row of the adjacency matrix ${\varvec{Y}}$, which represents the association of $lncRNA {l}_{i}$ with all miRNAs. $\left|{Y}_{i}\right|$ is the length of the vector ${Y}_{i}$, which is also the modules of the vector. $JSCMSNCP(i,j)$ represents the network consistency projection score of ${JSCM}_{j}$ on ${Y}_{i}$. Specifically, if the angle of the network projection of ${JSCM}_{j}$ on ${Y}_{i}$ is smaller, then the value of $JSCMSNCP(i,j)$ is larger.

With the integration of $JSCLSNCP(i,j)$ and $JSCMSNCP(i,j)$ calculated above, the final similarity score matrix can be integrated and normalized, as follows:

26 $$JSCSNCP\left(i,j\right)= \frac{JSCLSNCP\left(i,j\right)+ JSCMSNCP\left(i,j\right)}{\left|{JSCL}_{i}\right| + \left|{JSCM}_{j}\right|},$$

where, $JSCLSNCP(i,j)$ and $JSCMSNCP(i,j)$ represent the network consistency projection scores for $lncRNA {l}_{i}$ and $miRNA {m}_{j}$ in the lncRNA space and miRNA space, respectively. The $\left|\Delta \right|$ is a normalized operation to standardize the final prediction scores. Therefore, the value of $JSCSNCP(i,j)$ ranges between 0 and 1. Matrix ${\varvec{J}}{\varvec{S}}{\varvec{C}}{\varvec{S}}{\varvec{N}}{\varvec{C}}{\varvec{P}}$ is the final projection score matrix in lncRNA space and miRNA space, and each value in the matrix represents the final score of each lncRNA–miRNA pair. The final score is used to predict lncRNA with miRNA association. The higher the score, the higher the association.

## Results and discussion

### Self-performance evaluation of the JSCSNCP-LMA model

The performance evaluation of the JSCSNCP-LMA model was divided into two parts: the self-performance evaluation and the performance evaluation with other methods. For the self-performance evaluation, we verified the performance of JSCSNCP-LMA by using *k*-fold CV. We set the *k* values to 2,3,4,5, respectively, to perform the comparison test. In the k-fold CV scheme, 2272 known lncRNA–miRNA associations were divided into *k* equal subsets randomly. For each cross-validation experiment, *k *− 1 of them were used as the training set and the remaining one subset was used as the test sample. The predicted scores were calculated and sorted by JSCSNCP-LMA, the special ranking position was selected as the threshold value, and the offline area (AUC value) of the receiver operating characteristic (ROC) curve was used as a performance index to evaluate the prediction performance^67,68^. The ROC curve can plot the relationship between true positive rate (TPR) and false positive rate (FPR) at different thresholds. If the AUC is closer to 1, then the predicted performance is better. The TPR and FPR can be calculated as follows:

27 $$TPR=\frac{TP}{TP+FN},$$

28 $$FPR=\frac{FP}{FP+TN},$$

where, TP, FN, FP, and TN, each represent True Positive, False Negative, False Positive and True Negative.

**Figure 2:**
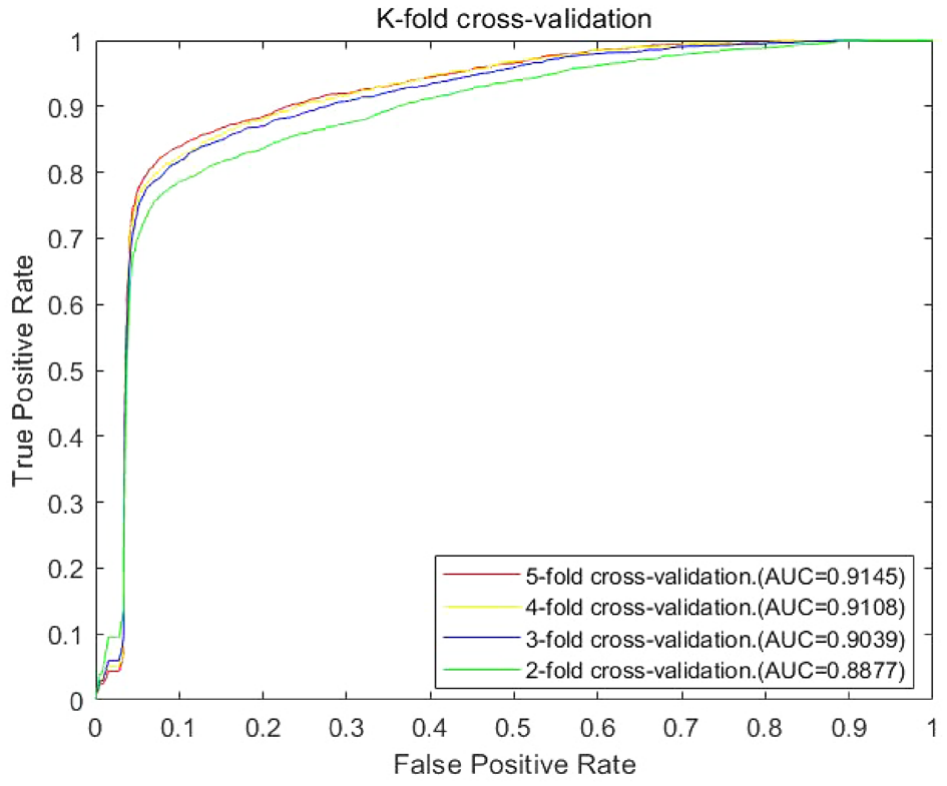
Influence of parameter variation on model prediction accuracy. The figure shows the ROC curve of k (where k = 2, 3, 4, 5) fold CV and the respective AUC values (fivefold CV: AUC = 0.9145; fourfold CV: AUC = 0.9108; threefold CV: AUC = 0.9039; twofold CV: AUC = 0.8877).

As shown in Fig. 2, it represented the ROC curves and AUC values under different *k* values in *k-*fold CV in Data 1, respectively.

**Figure 3:**
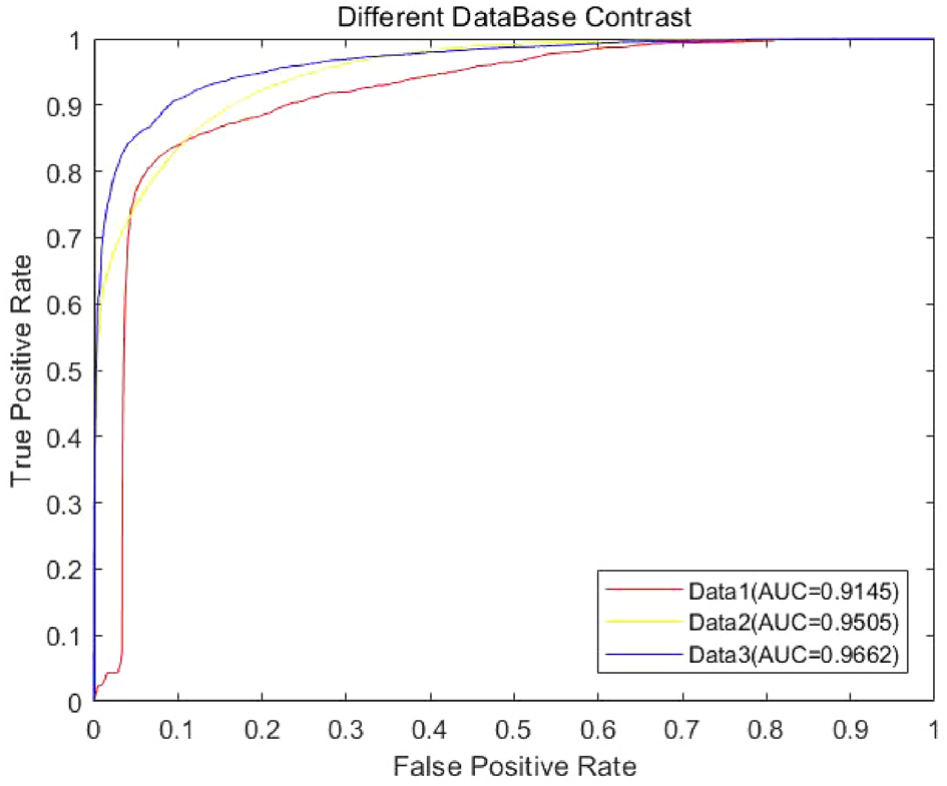
Prediction ability in different datasets. The figure shows the ROC curves in fivefold CV and the AUC values (Data 1: AUC = 0.9145; Data 2: AUC = 0.9505; Data 3: AUC = 0.9662).

To verify the generality of the method, we selected the data obtained from three different datasets by fivefold CV experiments to evaluate and compare its predictive analysis power. In the fivefold CV, 2272 lncRNAs–miRNAs associations in Data 1, 9086 lncRNAs–miRNAs associations in Data 2 and 8634 lncRNAs–miRNAs associations in Data 3 were included. For each cross-validation experiment, 4 of them were used as the training set and the remaining one subset was used as the test sample. The results of experiment are shown in Fig. 3.

**Figure 4:**
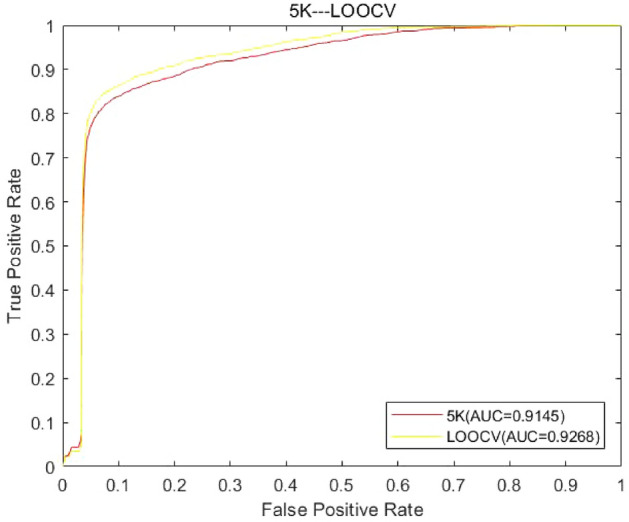
Prediction ability with different test methods. The figure shows the ROC curves in LOOCV and fivefold CV and the AUC values (LOOCV: AUC = 0.9268; fivefold CV: AUC = 0.9145).

To further verify the results of experiment, in this study, we used LOOCV and fivefold CV to compare their prediction performance on Data 1. The specific results are shown in Fig. 4.

**Figure 5:**
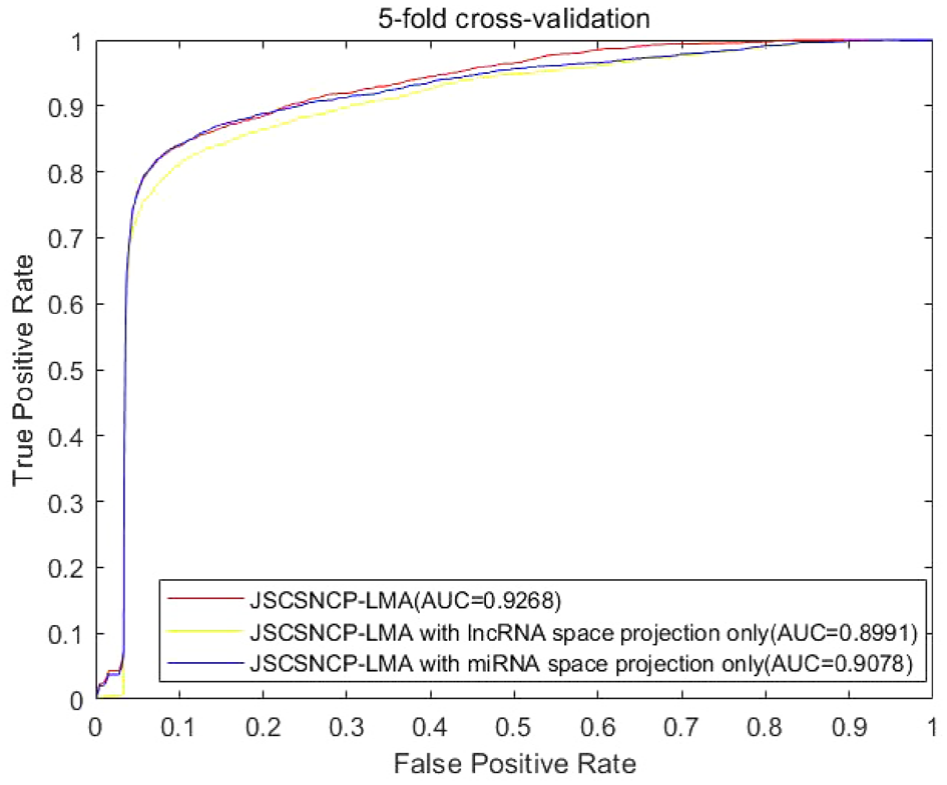
Influence of self-model’ s change on prediction accuracy. The figure shows that JSCSNCP-LMA has a reliable predictive performance with an AUC value of 0.9268. In the lncRNA projection space, the AUC value of JSCSNCP-LMA reach 0.8991. In the miRNA projection space, the AUC value is 0.9078. If the projection of two spaces is integrated, then the prediction performance can be greatly improved. Therefore, the JSCSNCP-LMA is reliable and has achieved good performance.

After comparing the results of different validation methods, we also considered the following conditions of the self-performance evaluation of JSCSNCP-LMA: (1) predictive performance with all information (JSCSNCP-LMA); (2) considering only the prediction performance of lncRNA space projection; (3) considering only the prediction performance of miRNA space projection. According to the above situation, the ROC curves and the AUC values in LOOCV on Data 1 are shown in Fig. 5.

Meanwhile, we also compared influence of self-model’ s change by using AUPR in Data 1, as shown in Table 2.

**Table 2:** Comparison of AUPR values for Influence of the change of model itself in fivefold CV.

Methods	AUPR
SSNCP-LMA	0.0730
JSSNCP-LMA	0.0715
JSCLSNCP-LMA	0.1501
JSCMSNCP-LMA	0.1591
JSCSNCP-LMA	0.1599

Where, SSNCP-LMA is method JSCSNCP-LMA only including self-tuning spectral clustering similarity algorithm. JSSNCP-LMA is method JSCSNCP-LMA only including self-tuning spectral clustering similarity algorithm and Jaccard similarity algorithm. JSCLSNCP-LMA is considering only the prediction performance of lncRNA space projection. JSCMSNCP-LMA is considering only the prediction performance of miRNA space projection.

### Comparison with the other methods

**Figure 6:**
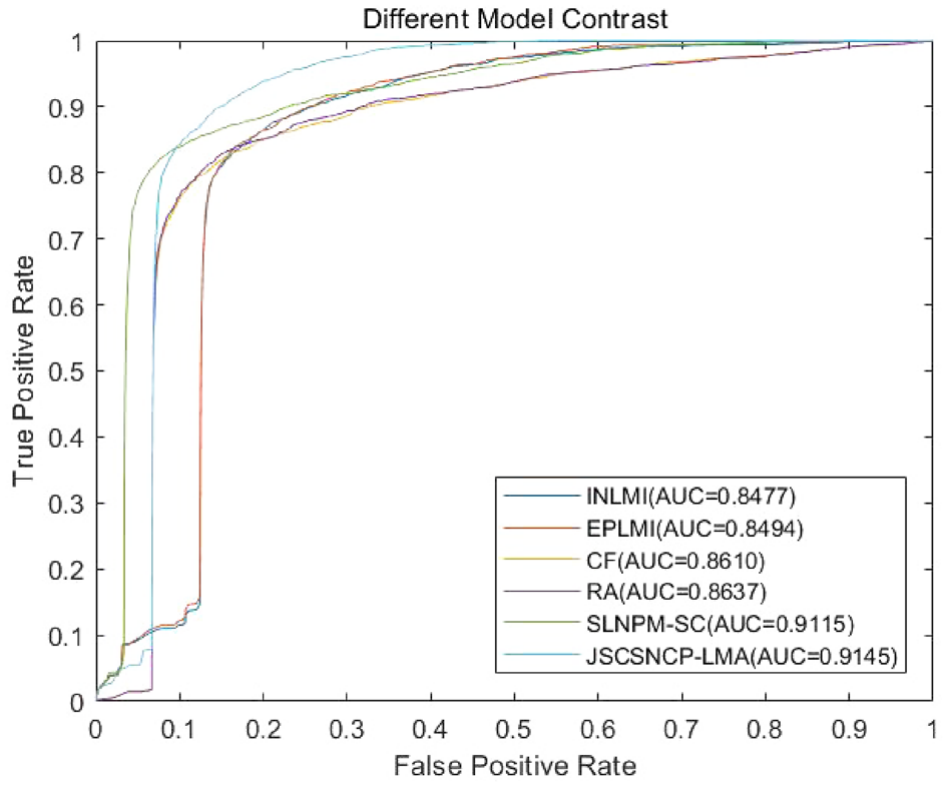
Prediction abilities with different models. The figure shows the ROC curves and AUC values of the six methods (INLMI, EPLMI, CF, RA, SLNPM-SC, JSCSNCP-LMA) by using fivefold CV.

To further verify the advantages of JSCSNCP-LMA, we used the known lncRNAs–miRNAs associations to compare JSCSNCP-LMA with other five methods. To the best of our knowledge, there were only a few machine-learning based methods for lncRNA–miRNA associations prediction. Here, we adopted EPLMI and INLMI^69^ as the benchmark methods. EPLMI is a two-way diffusion model which uses the known lncRNA–miRNA interaction-based bipartite graph and expression profiles to predict lncRNA–miRNA associations. We implemented EPLMI using its publicly available source code. INLMI integrates the expression similarity networks and the sequence similarity networks to predict lncRNA–miRNA associations. And we implemented this model according to descriptions in Ref.^69^. Since predicting lncRNA–miRNA associations could be considered as a link prediction task, we adopted several network link inference methods as baseline methods, i.e. the collaborative filtering method (CF)^63^ and the resource allocation algorithm (RA)^70^. In collaborative filtering method takes known lncRNA–miRNA interactions as a bipartite graph and exploits external information, such as expression profiles to calculate the lncRNA–lncRNA similarity and miRNA–miRNA similarity. Then, the CF method finds neighbors for each lncRNA and each miRNA, then uses the weighted average of its neighbor-interacting miRNA (lncRNA) to predict unknown associations, and then combines the lncRNA-based neighbor prediction and the miRNA-based neighbor prediction with a trade-off parameter. The resource allocation algorithm also formulates lncRNAs (miRNAs) as nodes and lncRNA–miRNA interactions as links in a bipartite graph. Interaction information is iteratively propagated from miRNAs to their linked lncRNAs, and then the information is allocated from lncRNAs back to miRNAs. After finite iteration, final resources for miRNAs are probabilities that the lncRNA interacts with these miRNAs. We used open source code to implement the method of sequence-derived linear neighborhood propagation (SLNPM) to predict the lncRNA–miRNA associations. Finally, we adjusted the parameters to achieve the best performance of each method. In this study, the fivefold CV was used to compare their prediction performance in Data 1. The results of the JSCSNCP-LMA comparison with the other methods are shown in Fig. 6. In Fig. 6, we can see that the AUC of the JSCSNCP-LMA model has a score of 0.9145. The proposed model performs much better than EPLMI (AUC score 0.8494), INLMI (AUC score 0.8477), RA (AUC score 0.8637), CF (AUC score 0.8610) and SLNPM-SC (AUC score 0.9115).

In addition, we also compared the model with the recent and more popular algorithms(LMI-INGI^71^ NDALMA^72^) by using data in Refs.^71,72^. The comparison results (AUC values) are shown in Table 3.

**Table 3:** Comparison of AUC values for different lncRNA–miRNA prediction methods in fivefold CV.

Methods	AUC
LncRNA-based CF	0.6359
KATZ	0.7439
MiRNA-based CF	0.8235
LFM	0.8253
EPLMI	0.8447
GBCF	0.8615
LncMirNet	0.8763
NDALMA	0.8810
LMI-INGI	0.8957
JSCSNCP-LMA	0.9154

### Case studies

To further investigate JSCSNCP-LMA model proposed by us for predicting lncRNA–miRNA associations, we constructed JSCSNCP-LMA based on the Data1 to predict unkown lncRNA–miRNA associations that didn’t include in the Data 1. After predicting, our prediction results had been verified by other databases or relevant literature. Therefore, we selected the top ten lncRNAs–miRNAs data pairs of the prediction scores. As shown in Table 4.

**Table 4:** Top 10 miRNAs-related candidate lncRNAs.

Rank	LNCRNAS	MIRNAS	CONFIRMED
1	RPll-244213.1	hisa-mir-497	starBase v2.0, lncRNASNP2
2	YLPMI	hisa-mir-195	-
3	LINC00649	hisa-mir-16	starBase v2.0, lncRNASNP2
4	RPll-329L6.1	hisa-mir-16	starBase v2.0, lncRNASNP2
5	LINC00649	hisa-mir-15a	starBase v2.0, lncRNASNP2
6	RP6-206I17.2	hisa-mir-195	starBase v2.0, lncRNASNP2
7	RPll-329L6.1	hisa-mir-497	starBase v2.0, lncRNASNP2
8	RP6-206I17.2	hisa-mir-16	starBase v2.0, lncRNASNP2
9	RPll-155D18.12	hisa-mir-15a	starBase v2.0, lncRNASNP2
10	RPll-155D18.12	hisa-mir-497	starBase v2.0, lncRNASNP2

Overall, 9 of the 10 data pairs of lncRNA–miRNA obtained by ranking prediction are confirmed by the corresponding database.

In addition, we also investigated the proportions of correctly predicted lncRNA–miRNA pairs among the top 100 highly scored predictions based on the bench marking dataset and compared their results with other methods. The results are shown in Table 5.

**Table 5:** Top 100 lncRNA–miRNA pairs prediction ratio.

Methods	Prediction ratio (%)
INLMI	84
EPLMI	85
CF	86
RA	86
SLNPM-SC	90
JSCSNCP-LMA	91

Therefore, we can argue that JSCSNCP-LMA can predict lncRNA–miRNA associations with high accuracy.

In addition, a number of studies have shown that lncRNA and miRNA play a key role in various diseases, especially cancer. To further evaluate the ability of JSCSNCP-LMA to predict potential lncRNA–miRNA associations, we conducted case studies on two common lncRNAs: ***H19***^73^ and ***HOTAIR***^74^. Among all the predicted results, we found and analyzed the top 15 miRNAs in ***H19*** and ***HOTAIR*** prediction score. In ***H19***, 14 miRNAs related to H19 have been verified by known databases or relevant literature. In ***HOTAIR***, 14 miRNAs related to HOTAIR have been also verified by known databases or relevant literature, as shown in Tables 6 and 7.

**Table 6:** The top 15 candidate miRNAs for *H19.*

Rank	LNCRNAS	MIRNAS	EVIDENCES	PMID
1	H19	hsa-mir-17	starBase v2.0	34041839
2	H19	hsa-mir-106a	starBase v2.0	30993766
3	H19	hsa-mir-20b	starBase v2.0	31894264
4	H19	hsa-mir-130b	starBase v2.0	29744254
5	H19	hsa-mir-106b	starBase v2.0	34041839
6	H19	hsa-mir-130a	starBase v2.0	33616375
7	H19	hsa-mir-519d	starBase v2.0	25366760
8	H19	hsa-mir-301b	starBase v2.0	30625468
9	H19	hsa-mir-93	starBase v2.0	31953562
10	H19	hsa-mir-20a	starBase v2.0	30092355
11	H19	hsa-mir-301a	starBase v2.0	30814872
12	H19	hsa-mir-454	starBase v2.0	30809286
13	H19	hsa-mir-19b	starBase v2.0	28765931
14	H19	hsa-mir-19a	starBase v2.0	28765931
15	H19	hsa-mir-302e	No	27075472

**Table 7:** The top 15 candidate miRNAs for HOTAIR.

Rank	LNCRNAS	MIRNAS	EVIDENCES	PMID
1	HOTAIR	hsa-mir-519d	starBase v2.0	25366760
2	HOTAIR	hsa-mir-130b	starBase v2.0	29744254
3	HOTAIR	hsa-mir-17	starBase v2.0	33750300
4	HOTAIR	hsa-mir-20a	starBase v2.0	29740493
5	HOTAIR	hsa-mir-106a	starBase v2.0	30993766
6	HOTAIR	hsa-mir-454	starBase v2.0	28182000
7	HOTAIR	hsa-mir-301b	starBase v2.0	29744254
8	HOTAIR	hsa-mir-130a	starBase v2.0	33616375
9	HOTAIR	hsa-mir-20b	starBase v2.0	30468285
10	HOTAIR	hsa-mir-301a	starBase v2.0	30814872
11	HOTAIR	hsa-mir-106b	starBase v2.0	33773548
12	HOTAIR	hsa-mir-93	starBase v2.0	32144238
13	HOTAIR	hsa-mir-19b	starBase v2.0	34249429
14	HOTAIR	hsa-mir-19a	starBase v2.0	–
15	HOTAIR	hsa-mir-302d	No	–

In recent years, ***H19*** has become a research hotspot due to its ectopic expression in human diseases, especially malignant tumors, and plays an important role as an oncogene in human malignant tumors. Meanwhile, ***H19*** has been shown to be involved in the development and malignant progression of many tumors, and promote cell growth, invasion, migration, epithelial-mesenchymal transition, metastasis and apoptosis. In addition, ***H19*** can isolate some miRNAs, promote multi-layer molecular regulatory mechanisms, or co-affect the occurrence of some diseases with some miRNAs. For example, in Table 3, Qin et al.^75^ verified that both ***H19*** and ***hsa-mir-301b*** were prognostic factors of cervical cancer. Luo et al.^76^ verified that ***H19*** played an important role in regulating inflammatory processes in retinal endothelial cells by regulating ***hsa-mir-93*** under high-glucose condition. He et al.^77^ verified that ***H19*** and ***hsa-mir-17/hsa-mir-106b*** could affect the treatment efficacy of patients with chronic hepatitis B. Zhao et al.^78^ verified that ***H19*** could regulate the expression of ID2 through competitive binding to ***hsa-mir-19a/b***, which played a role in the proliferation of acute myelocytic leukemia (AML) cells. Moreover, ***H19*** also plays an important role in the generation or treatment of other cancers, such as colon cancer, breast cancer, lung cancer and prostate cancer^79–82^. Similarly, there are also many miRNAs that play an important role in the generation or treatment of cancer. For example, Rafael Sebastian Ford et al.^83^ verified the oncogenic role of ***hsa-mir-130b*** in prostate cancer. However, no biological researchers have directly verified the association between ***H19*** and ***hsa-mir-130b*** at present. Therefore, the prediction results provided by the JSCSNCP-LMA are mainly used to provide biological researchers with research directions, so as to improve the efficiency of biological research.

Like ***H19***, ***HOTAIR*** is one of the most widely studied abnormally regulated lncRNAs in human cancers. Studies have shown that in preclinical cancer research^84^, ***HOTAIR*** can control basic biochemical and cellular processes and promote proliferation, invasion, survival, drug resistance and metastasis through interactions with a variety of other biological factors. And ***HOTAIR*** has been also shown to promote tumor progression by regulating miRNAs expression and function. For example, in Table 4, Pan et al.^85^ verified that the regulatory mechanism between ***HOTAIR*** and ***hsa-mir-17*** played an important role in ruptured intracranial aneurysm disease. Cao et al.^86^ verified that the regulatory mechanism between ***HOTAIR*** and ***hsa-mir-20a*** played an important role in liver cancer cells. Bao et al.^87^ verified that ***HOTAIR*** could affect human chondrosarcoma disease by controlling ***mir-454-3p***. Moreover, ***HOTAIR*** also plays an important role in the generation or treatment of other cancers, such as lung cancer, rectal cancer, prostate cancer and cervical cancer^88,89^. Similarly, there are also many miRNAs that play an important role in the generation or treatment of cancer. For example, Liu et al.^90^ verified that ***hsa-mir-106b*** also played a crucial role in rectal cancer. Therefore, it is very important to study the unknown lncRNA–miRNA associations.

## Conclusions

LncRNAs–miRNAs associations are critical to many biological activities and are closely related to the development of various diseases. Therefore, exploring and identifying these associations can help to understand the function of lncRNAs/miRNAs and complex disease mechanisms. In this paper, we proposed a prediction method named the JSCSNCP-LMA that was different from other traditional methods. JSCSNCP-LMA achieved excellent prediction performance by using Jaccard similarity algorithm, self-tuning spectral clustering similarity algorithm, cosine similarity algorithm and known lncRNA–miRNA association networks. JSCSNCP-LMA did not require redundant parameters and showed a obvious advantage when the known experimentally validated lncRNA–miRNA associations were insufficient. To validate the predictive performance of the JSCSNCP-LMA, LOOCV and fivefold CV were used. Results showed that the proposed method outperformed other methods and effectively identified potential lncRNAs–miRNAs associations. Meanwhile, the prediction capabilities of proposed models were also tested by case studies. We validated the results of case studies by using known literature and datasets. In conclusion, JSCSNCP-LMA is promising for lncRNA–miRNA association prediction. It not only has a good performance, but also has good expansibility. For example, metabolite-disease association prediction^91^, miRNA-disease association prediction^92^, lncRNA-disease association prediction^93^, and lncRNA-protein association prediction^94^.

Although this model has achieved good results, it still has some limitations. First of all, the known association data between lncRNAs and miRNAs is relatively small, which will affect the final prediction results. What’s more, this model only uses a single lncRNAs–miRNAs association data and does not combine other association data, such as lncRNAs-diseases, miRNAs-diseases and lncRNAs-proteins. This may lead to inaccurate prediction results due to the failure to consider the influence of other factors. Finally, in the algorithm, we only simply compare the influence of the default parameters on the algorithm and do not use the optimization algorithm to find the optimal solution automatically. This will reduce the accuracy of the prediction results. In order to better reduce the prediction bias and improve the prediction performance, our future work mainly focuses on the optimization of similarity calculation and method fusion. At the same time, we will also build an algorithm to automatically seek the optimal parameters, so as to improve the accuracy of the prediction results. Finally, we will try to combine other association data to consider the predicted results from multiple perspectives. We believe that when more biological knowledge is applied to a refined fusion method, the accuracy of model prediction can be improved. And our method can be helpful for relevant biomedical research.

## Supplementary Information

Supplementary Information.

## Data Availability

miRBase: http://www.mirbase.org/index.shtml. miRmine: http://guanlab.ccmb.med.umich.edu/mirmine. NONCODE: http://www.noncode.org. lncRNASNP: http://bioinfo.life.hust.edu.cn/lncRNASNP. ENCORI: http://starbase.sysu.edu.cn/.
