# Supplementary material for: JSCSNCP-LMA: a method for predicting the association of lncRNA–miRNA
Source: Sci Rep. 2022 Oct 11;12:17030. doi: 10.1038/s41598-022-21243-y (PMC9552706; doi:10.1038/s41598-022-21243-y)
Supplement: Supplementary file 1 — Supplementary Information. [file 41598_2022_21243_MOESM1_ESM.zip › test data/Data Availability.docx]

# Data Availability

miRBase：<http://www.mirbase.org/index.shtml>

miRmine：http://guanlab.ccmb.med.umich.edu/mirmine

NONCODE：<http://www.noncode.org>

lncRNASNP：<http://bioinfo.life.hust.edu.cn/lncRNASNP>

ENCORI ：http://starbase.sysu. edu.cn/
